# Effect of Restraint Stress during Gestation on Pentylenetetrazol-Induced Epileptic Behaviors in Rat Offspring

**Published:** 2013-09

**Authors:** Pariya Hashemi, Logman Ebrahimi, Ehsan Saboory, Shiva Roshan-Milani

**Affiliations:** 1 Department of Physiology, Medical Faculty, Urmia University of Medical Science, Urmia, Iran; 2 Department of Physiology, Neurophysiology Research Center, Urmia University of Medical Science, Urmia, Iran

**Keywords:** Epileptic behavior, Gestation, Pentylenetetrazol, Rat, Restraint stress

## Abstract

***Objective(s):*** Epilepsy is a neurodevelopmental disorder which is strongly influenced by genetic and environmental factors. Gestational stress has been shown to be an important factor for affecting seizure susceptibility. The present study was conducted to address whether gestational stress may affect pentylentetrazol (PTZ)-induced epileptic behavior in rat offspring in a sex- and age- dependent manner.

***Materials and Methods:*** Pregnant rats were divided into control and stressed groups (n=6 in each). In the stressed group, pregnant rats were under restraint stress and held immobile in the Plexiglas tube twice per day one hour per session for three consecutive days started on day 17 of pregnancy. To induce seizure, on postnatal days 15 (P15) and 25 (P25), PTZ (40-50 mg/kg, IP) was injected to rat offspring (n=12, one male and one female from any litter for each group/day). Then, epileptic behaviors of each rat were recorded.

***Results:*** Epileptic behaviors of stressed pups showed significant changes in comparison to control ones. The time to onset of the first epileptic behavior was shortened while mean duration and frequency of tonic-clonic attacks increased in stressed pups on both P15 and P25. Female offspring were different from male offspring in terms of epileptic behavior. Moreover, focal attacks were more obvious and significantly longer in the offspring of stressed group at the age of 25 days than those of 15 day old.

***Conclusion:*** Prenatal restraint stress potentiated PTZ-induced epileptic behavior, age and sex dependently, probably due to alteration of neural and endocrine pathways during developmental process. Male and younger rats were more sensitive to stress than female and older ones.

## Introduction

Epilepsy is a common neurological disorder that affects individuals of all ages. Despite all research advancements, some etiologic and preventive aspects of epilepsy have not been completely established yet, which represent a significant need for research. During gestation, development of central nervous system (CNS) depends on interactions between genetic and environmental epigenetic factors, some of which could affect susceptibility of the developing brain to epilepsy (1). The complex interactions of prenatal environmental factors with the developing brain suggest that it has multiple site- and time-specific vulnerabilities that may contribute to the pathogenesis of several forms of epilepsy. On the other hand, it may provide several new forms of prevention. From this respect, it has been suggested that prenatal environmental factors such as stress in animals exert a profound influence on development of nervous system and can affect susceptibility to epilepsy later in life (2, 3). In adult humans, it has been also reported that stress aggravates seizures in epileptic patients and may even stimulate acute seizures in persons with no history of seizures. Therefore, procedures to reduce stress have been proposed as a way to improve seizure control (4). Although stress affects seizure susceptibility in animals and humans, the underlying mechanisms remain unclear (5). The major pathway applied in synchronizing consequences of stress in most mammalian species is hypothalamic-pituitary-adrenal (HPA) axis (6) and stress can stimulate activation of HPA axis and impair feedback regulation of this axis. Stress and elevated levels of cortisol, in turn, may affect neuronal excitability, increase susceptibility for seizures and contribute to a damaging course of epilepsy (7). 

Prenatal stressors can similarly alter regulation of the HPA axis and affect seizure susceptibility in infancy and afterwards. However, precise effect of corticosteroids and gestational stress on seizure vulnerability is not enough clear (3). Previously, our research group demonstrated that prenatal stress increased susceptibility to seizures in pilocarpine–induced epilepsy model in male and female rats (2, 3). However, the effect of stress on epilepsy is controversial; a number of reports have documented findings contradictory to those previously described in the literature. Accordingly, some experimental stressors including swim stress have been shown to elicit anticonvulsant effects using hippocampal–enthorinal combined slices (5). Thus, both pro-convulsant (2, 3, 8, 9) and anticonvulsant (5) effects of stress have been reported, depending on the experiment conditions. The appearance of such changes depends on timing of maternal stress, its intensity and duration, gender of the offspring and experimental models of both epilepsy and stress (10). Pentylenetetrazol (PTZ), a noncompetitive GABA_A _receptor antagonist, is a convulsant-inducing chemical agent which is widely used in experimental models of seizure (11-13). Moreover, there is little knowledge about age- and sex-dependent influence of prenatal stress on epileptic behavior, especially over different models of epilepsy. Therefore, this study was designed to investigate impact of prenatal restrain stress on epileptic behaviors induced by PTZ to assess whether prenatal stress has more general facilitating influence on other forms and models of seizure activities.

## Materials and Methods

***Ethical approval***

All of the experimental protocols and procedures were followed according to guidelines of the 1975 Declaration of Helsinki, as reflected in the guidelines of the Medical Ethics Committee, Ministry of Health, Iran. In addition, Regional Medical Ethics Committee in the West Azarbayjan Province, Iran approved this study. 

***Subjects***

Ten-week-old male and female Wistar rats (200–250 g) were obtained from the animal facility, Urmia University of Medical Sciences, Urmia, Iran. The rats were housed in groups of 4 per cage under a 12 hr light/dark cycle (07:00 to 19:00 lights were on) at 22±2°C with free access to food and water. All female rats were mated at 12 weeks with a sexually experienced male of the same genotype. Each female was paired with one male at 09:00 and was checked for plugs at 15:00. If a plug was present, the female rat was immediately moved to a new cage, where she remained in isolation for the entire gestation period. If no plug was observed, the animal was returned to her home cage for a new mating chance. Pregnant rats were divided into two control and stress groups (n= 6 in each).

***Restraint stress procedure***

Pregnant rats in the stressed group were exposed to the stressor on gestation days 17, 18 and 19 (E17, E18 and E19, respectively, late gestation stress). For restraint stressed rats, stress involved transporting from the home cage to the experimental room and placing the pregnant female in a restraint chamber (a transparent, plastic, cylindrical chamber, 6 cm in diameter and 16 cm in length) under normal room conditions. The animals were restrained for 60 min twice per day (between 08:00–11:00 and 15:00–18:00) for 3 consecutive days. This protocol has previously been shown to cause alterations in the regulation of HPA (hypothalamic-pituitary-adrenal) axis in the offspring (8, 14). In the control group, pregnant females were transported to the experimental room on E17, E18 and E19 and were handled similar to the stressed groups, except for stressor exposure.

***Body weight measurement***

After parturition, the pups in each litter (control and stressed) were counted and weighted at 09:00 on the first postnatal day (P1). The weight of each pup was recorded again at 09:00 on P6, P15 and P25.

***Behavioral assessment***

On P15 and P25, PTZ (40-50 mg/kg) was injected intraperitoneally (IP) to the offspring of each group. One male and one female pup from each litter were assigned to each experimental day (n=12 for each group for P15 and P25). Following the injection, behavior of each rat was observed and documented for 90 min by a digital camera. The seizure rating was assessed using a previously defined scale (15): 0=normal; 1=immobilization, sniffing; 2=head nodding, facial and forelimb clonus (short myoclonic jerk); 3= continuous myoclonic jerk, tail rigidity; 4=generalized limbic seizures with kangaroo posture or violent convulsion; 5=continuous generalized seizures (tonic or clonic-tonic convulsions).

***Statistical analyses***

The results were expressed as mean ± SEM. The data on normally distributed weights were analyzed using t-test. Behavioral assessment data that were not normally distributed were analyzed by Mann–Whitney U-test and Kruskal–Wallis one-way ANOVA. The data related to mortality rate and percentage of tonic-clonic seizure was analyzed using Fisher's exact test. The results with *P*< 0.05 were considered significant. 

**Table 1:** Classification of seizure parameters in 15 and 25-day-old rats prenatally exposed to restraint stress after administration of PTZ (40-50 mg/kg, IP

P- value	P25		P15		Epileptic Behaviors
	(RS)	(C)	(RS)	(C)	
P=0.0003, C vs. RS ( P15) P=0.004, C vs. RS ( P25)	93.08 ± 17.8 15.25 ± 6.06		78.5 ± 16.58 18.08 ± 4.1		Time to onset of first epileptic behavior (S)
P=0.03, C vs. RS ( P15 & P25)	3 ± 0.3 3.6 ± 0.4		2.5 ± 0.3 4 ± 0.5		Number of immobilization, sniffing
P>0.05, C vs. RS ( P15 & P25) P=0.02, P15 vs. P25 (RS)	4.5 ± 0.9 6.3 ± 0.8		4.9 ± 0.7 5.4 ± 0.9		Duration of immobilization, sniffing (min)
P=0.02, C vs. RS ( P15) P=0.0005, C vs. RS ( P25) P=0.03, P15 vs. P25 (RS)	1.6 ± 0.28 3.75 ± 0.39		1.25 ± 0.25 2.5 ± 0.4		Number of short myoclonic jerk
P=0.0001, C vs. RS ( P15) P=0.008, C vs. RS ( P25)	0.97 ± 0.24 2.7 ± 0.5		0.87 ± 0.2 3.6 ± 0.3		Duration of short myoclonic jerk (min)
P=0.01, C vs. RS ( P15 & P25) P=0.006, P15 vs. p25 (RS)	0.33 ± 0.1 1.16 ± 0.24		0.08 ± 0.08 0.25 ± 0.25		Number of focal seizure
P =0.02, C vs. RS ( P15 & P25) P =0.006, P15 vs. p25 (RS)	0.1 ± 0.04 0.5 ± 0.13		0.034 ± 0.03 0.16 ± 0.1		Mean dration of focal seizure (min)
P =0.01, C vs. RS ( P15 & P25)	0.33 ± 0.14 1 ± 0.3		0.08 ± 0.08 3 ± 0.94		The number of tonic-clonic seizure
P =0.01, C vs. RS ( P15) P >0.05, C vs. RS ( P25)	0.09 ± 0.042 1.56 ± 0.54		0.01 ± 0.01 2.55 ± 0.88		Mean duration of tonic-clonic seizure (min)
P>0.05, Fisher Exact Test	0% 8%		0% 0%		Mortality rate during attacks

C, control; RS, restraint-stressed; P15, postnatal day 15; P25, postnatal day 25

**Figure 1:**
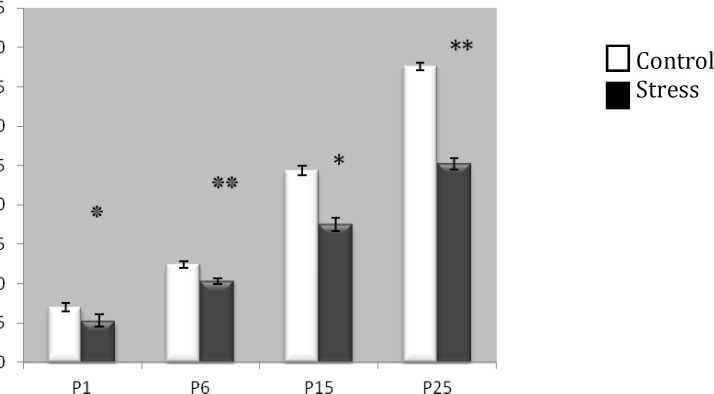
The effect of exposure to restraint stress during gestation on offspring weight at different time points. Restraint stress leads to low birth weight, which continues to postnatal day 25 (P25). The weight in all time points was significantly lower in stressed animals compared with the control group (*P<0 .05*) ٭ C vs RS (P1); ٭٭C vs RS (P6); *C vs RS (P15); **C vs RS (P25

## Results

***Effects of gestational stress on body weight of rat offspring***

Significant differences in litter body weight were detected between experimental groups at different times. In restraint stressed pups, mean body weight of the offspring on birth and postnatal days 6, 15 and 25, significantly decreased in comparison to the control pups (*P< *0.05). Effect of restraint stress on pup body weight at various time points is illustrated in Figure 1.

***Effects of gestational stress on PTZ-induced epileptic behavior in rat offspring***

Following the IP administration of PTZ 40-50 mg/kg to rat offspring in both groups, all the animals displayed comparable seizure activities on P15 and P25. Behavioral changes were observed for 30-60 sec after PTZ injection and scored according to the 1–5 rating scale. In spite of the comparable seizure scale in experimental groups, statistical analyses revealed significant differences in characteristics of epileptic behaviors. On P15, time to onset of the first epileptic behavior was 78.5±16.58 s in control pups, which decreased significantly to 18.08±4.1 sec in stressed pups (*P=*0.0003); on P25, this time was 93.08±17.8 sec in control pups, which significantly decreased to 15.25 ± 6.06 sec in stressed pups (*P= *0.004*)*. In addition, on P15, percentage of offspring in each group that had a tonic–clonic seizure was 8% (1 of 12) in control pups while this percentage significantly increased to 58% (7 of 12) in stressed pups (*P=*0.02, Fisher's Exact Test). On P25, 33% (4 of 12) of the offspring showed tonic-clonic seizures in control group whereas, in stress group, 42% (5 of 12) of them demonstrated tonic-clonic seizures. Moreover, in each animal, the number (or mean frequency) of tonic-clonic attacks increased from 0.08±0.08 in control pups to 3±0.94 in stressed pups on P15; in the same way, it increased from 0.33±0.14 in control pups to 1± 0.36 in stress group on P25. Furthermore, mean duration of tonic-clonic attacks significantly increased from 0.01± 0.01 min in the control pups to 2.55±0.88 min in the stressed pups on postnatal day 15. On P25, mortality rate was 8% during attacks in the stressed pups whereas no mortality was observed in the control group. The effects of prenatal stress on PTZ-induced seizure behaviors are summerized in Table 1.

**Table 2:** Classification of sex differences in PTZ-induced seizure parameters in rat offspring, prenatally exposed to restraint stress

Epileptic behavior	Sex	C ,P15	RS, P15	C, P25	RS, P25	P -value
Time to onset (Sec)	M	*64.5±18.9	*24.33±6.4	123.33±24.9	24.3±6.1	*P=0.03
	F	92.5±27.8	*11.83±4.6	62.8±20.3	*11.8±4.6	*P=0.04
Number of focal seizure	M	*0.33±0.2	0.5±0.5	0.5±0.22	1±0.36	*P =0.02
	F	0±0	0±0	0.16±0.16	*1.5±0.8	*P =0.02
Duration of focal seizure(min)	M	*0.06±0.06	0.33±0.33	0.1±0.05	0.53±0.2	
	F	0±0	0±0	0.04±0.04	*0.58±0.1	
Number of T-C seizure	M	0.16±0.16	3.5±1.5	0.5±0.22	1.16±0.65	
	F	0±0	1.8±1.1	0.16±0.16	0.83±0.4	
Duration of T-C seizure (min)	M	0.02±0.02	2.05±0.8	0.16±0.07	1.9±0.98	
	F	0±0	3.04±1.6	0.01±0.01	1.1±0.54	

M, male; F, Female; C, control; RS, restraint-stressed; P15, postnatal day 15; P25, postnatal day 25; T.C, Tonic-Clonic seizure

***Age- and sex-dependent effects of gestational stress on PTZ-induced epileptic behavior in 15 and 25 day old rats***

In order to compare age- and sex-dependent differences between experimental groups, statistical analyses were performed using Kruskal–Wallis nonparametric ANOVA. According to the observation and analysis, focal seizures were more frequent and longer in 25 day old rats in comparison to 15 day old rats, in both control and stress groups. However, tonic-clinic seizures were getting worse, lasting longer and happening more often in 15 than 25 day old rats. Although these age-dependent differences were observed in both control and stress pups, significant changes were only found in stress pups (Table 1). 

In spite of some differences between male and female rats in experimental groups, statistical analysis revealed no significant changes in most epileptic parameters.  Although more frequent and longer tonic-clonic seizures were observed for male offspring in stress groups, the differences were not significant (Table 2). 

## Discussion

Main finding of the present study was that prenatal exposure to stress can decrease body weight and enhance epileptic behaviors in newborn rat offspring. In this study, pregnant rats were exposed to restraint stress on gestation days 17, 18 and 19 and then their pups were examined for PTZ-induced epileptic behavior on P15 and P25. According to the results, the time to onset of epileptic behavior was shortened while duration and frequency of tonic-clonic seizure(s) and percentage of generalized tonic-clonic convulsions significantly increased in the rats which were prenatally exposed to restraint stress. We previously reported that prenatal restraint (2-3) and predator stresses (2) were associated with pro-convulsant effects in seizures induced by pilocarpine. In the present study, the impact of prenatal restrain stress on epileptic behaviors induced by PTZ was investigated to assess whether prenatal stresses might have a more general facilitating influence on other forms and models of seizure activities in an age- and sex dependent manner. 

It has been expressed that stress in gestational period probably leads to changes in cerebral maturation and causes abnormalities in neuronal correlations, which increases susceptibility to cerebral function disorders such as epilepsy (16, 17). In this respect, prenatal stress produces learning deficits associated with inhibition of neurogenesis (18) or decline in neuronal size  (19) in the hippocampus and decreases seizure threshold (8, 17). It has been also suggested that long-lasting effects of stress have been related to disturbance in the function of HPA axis (20) and alteration of its feedback regulation, which in turn, causes higher basal secretion of corticotrophin releasing factor (CRF). CRF and glucocorticoids show pro-convulsant effects in pups and are known to decrease seizure threshold and cause alterations in fetal central nervous systems (21-24). It was previously shown that prenatal predatory (2) and restraint (2, 3) stress increased corticosterone blood levels in rat offspring, which was consistent with the above mentioned findings. 

It is known that GABA_A_ receptors have a prominent role in mediating tonic inhibition, particularly in hippocampal pyramidal cells (25). This tonic inhibition is responsible for generating nearly 75% of total inhibitory charge received by hippocampal neurons (26). PTZ is a noncompetitive antagonist that blocks GABA-mediated Cl-influx and leads to neuronal depolarization and consequently propagation of seizures. The present results showed that acute exposure to PTZ could produce lethality with a very narrow range of lethal and convulsive dose. Because of the extremely narrow margin between PTZ lethal and convulsive doses, PTZ was used at a convulsive dose of 40-50 mg/kg, which was very close to its sub-convulsive dose (27). At this dose, it was not possible to determine precise PTZ-induced epileptic scales as well as its mortality rate. In addition, effects of prenatal stress on PTZ-induced epileptic behavior were not completely compared with other epileptic models such as pilocarpine in terms of scale, severity of epilepsy, time course and mortality rate. Despite these limitations, the observation that prenatal restraint stress potentiated epileptic behavior in both models and produced almost similar phenotype of alteration, suggested that prenatal stress might target a common mechanism, encoding facilitation of epileptic behaviors in these models. While it was hypothesized that this could be an alteration in the function of HPA axis, further effort is required to establish precise nature of their endocrine or neurochemical interaction.

The present study of mixed physical-social (prenatal restrain) stress showed important age and sex-related differences in behavioral epilepsy. Seizures were dominated by focal attacks in the offspring at the age of 25 days whereas tonic-clonic attacks were mainly dominant at the age of 15 days in both control and stress groups (Table 1). Therefore, although it seems that developmental differences, independent from stress, affect epileptic behaviors, the observation that significant changes only occurred in stress groups suggested that prenatal stress could potentiate age-dependent alteration of neuronal maturation. In a similar manner, more frequent and longer tonic-clonic seizures were observed for the male offspring in stress groups, indicating that male rats were more sensitive to stress than female ones (Table 2). Although statistical analysis did not detect significant changes in some parameters, probably due to small sample size (n=6 for male and female rats), influence of sex on epileptic behavior was clear and could not be ignored. According to previous studies, men and women may respond differently to stressors (28). Subtle sex differences have been also documented in several animal models of stress (29-30). Also, effects of chronic stress on seizure risk and GABA_A_ receptors were shown in rats (28), which suggested that differences in activity among sex hormones could differentially influence seizures and susceptibility of HPA axis in male and female rats (31). 

Altogether, since prenatal restraint stress and other stress models (2, 8, 21) have a facilitating effect on epilepsy, controlling stress factors during gestation may represent a potential target in developing novel ways for epilepsy control.

## Conclusion

Results of the present study revealed an age- and sex-dependent impact of prenatal stress on epileptic behavior in rat offspring. Undoubtedly, there are many neural and endocrine pathways, through which stress can alter brain function and thereby affect seizure susceptibility. Studying the effect of prenatal stresses on vulnerability to epilepsy may create an opportunity for improving prevention or suppression of epilepsy.
